# Supplementary figures and images for: Variable and conserved features of copy-back viral genome populations generated de novo during Sendai virus infection
Source: J Virol. 2026 Jan 9;100(2):e01805-25. doi: 10.1128/jvi.01805-25 (PMC12911868; doi:10.1128/jvi.01805-25)

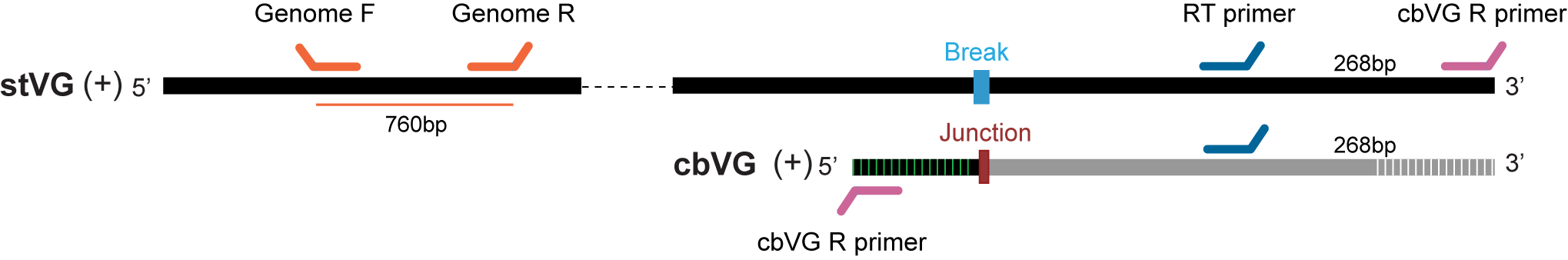

Supplement: Fig. S1 — cbVG-PCR schematic. [file jvi.01805-25-s0001.tif]

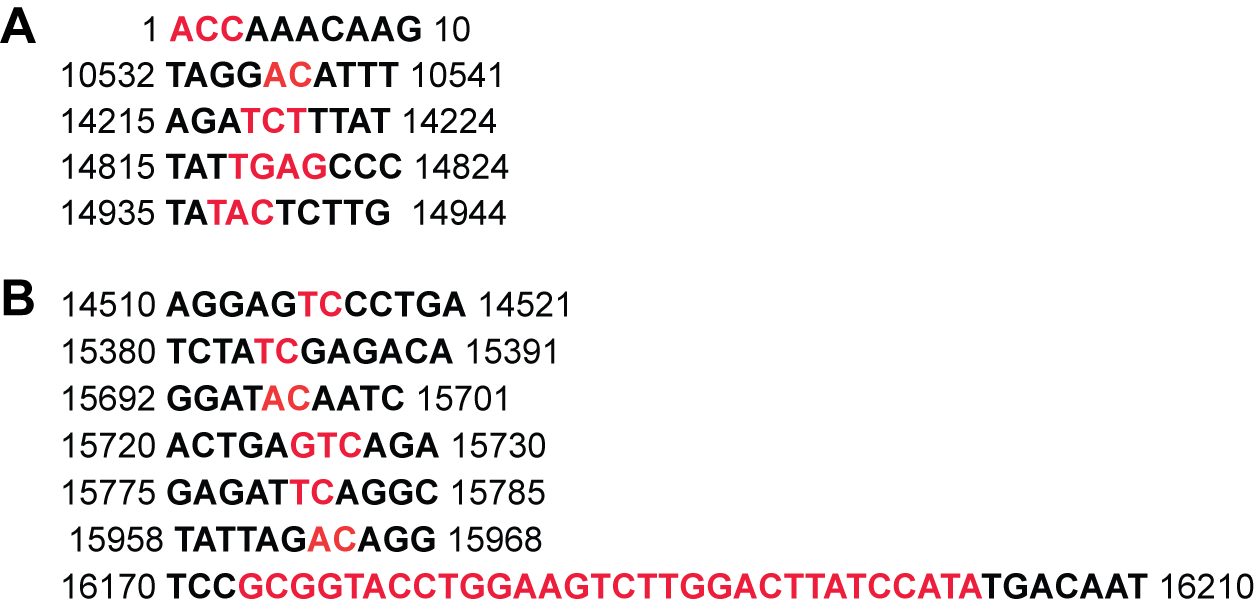

Supplement: Fig. S2 — Sequences flanking shared break or rejoin positions. [file jvi.01805-25-s0002.tif]

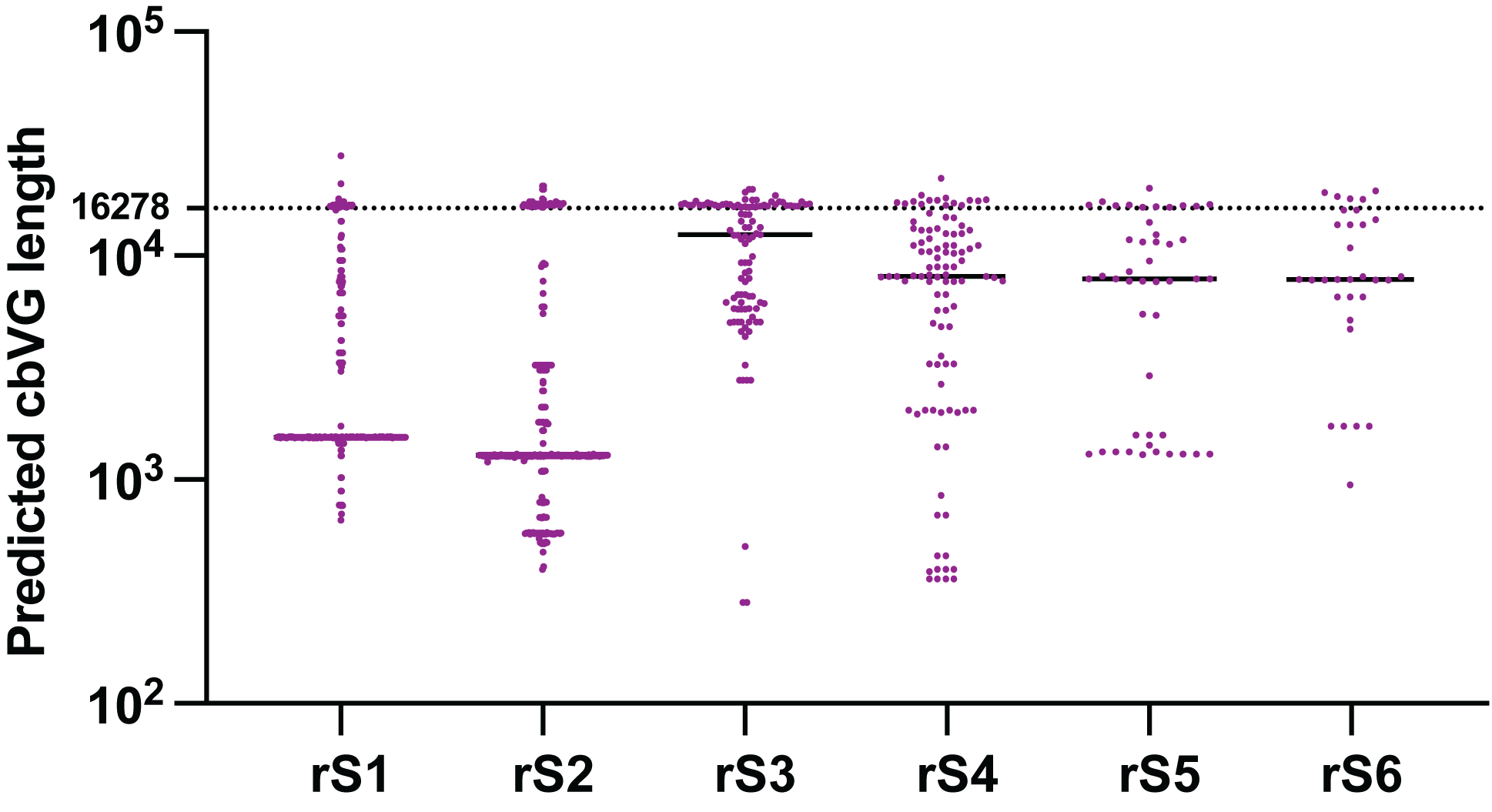

Supplement: Fig. S3 — Predicted length of all unique cbVGs. [file jvi.01805-25-s0003.tif]
