# Supplementary material for: Variable and conserved features of copy-back viral genome populations generated de novo during Sendai virus infection
Source: J Virol. 2026 Jan 9;100(2):e01805-25. doi: 10.1128/jvi.01805-25 (PMC12911868; doi:10.1128/jvi.01805-25)
Supplement: Supplemental material — Tables S1 and S2; legends Figures S1 to S3. [file jvi.01805-25-s0004.docx]

**Figure S1: cbVG-PCR schematic.** Diagram of the standard viral genome (stVG) and a representative copy-back viral genome (cbVG) of unknown length. The break position of the cbVG is indicated with light blue on the stVG, and the junction rejoin site is marked with burgundy on the cbVG, with the complementary regions represented with black/green stripes and grey/white stripes. The blue RT primer is located 268 bp from the 3’ end of the antigenome. The pink primer represents the reverse primer for cbVG detection, while the orange primers correspond to genomic PCR primers. The orange line below indicates the expected PCR product length of 760 bp.

**Figure S2: Sequences flanking shared break or rejoin positions.** (A) Sequences surrounding shared break sites. The nucleotides at the break position are shown in red, and the flanking nucleotides are shown in black. (B) Sequences surrounding shared rejoin sites. The nucleotides at the rejoin position are shown in red, and the flanking nucleotides are shown in black.

**Figure S3: Predicted length of all unique cbVGs.** cbVGs and their predicted length for each high-MOI passaged P3 stock. Each dot represents a unique cbVG. The black horizontal lines indicate the median cbVG length for each stock. The dashed line indicates the length of the standard genome.

**Table S1: Dominant cbVG junctions of each P3 high-MOI passaged stock.**

| **rS1** | | | **rS2** | | | **rS3** | | | **rS4** | | | **rS5** | | | **rS6** | | |
| --- | --- | --- | --- | --- | --- | --- | --- | --- | --- | --- | --- | --- | --- | --- | --- | --- | --- |
| Break_Rejoin | Length | % | Break_Rejoin | Length | % | Break_Rejoin | Length | % | Break_Rejoin | Length | % | Break_Rejoin | Length | % | Break_Rejoin | Length | % |
| 14821_16189 | 1548 | 29.6 | 15096_16178 | 1284 | 18.5 | 12412_15100 | 5046 | 33.4 | 12827_15021 | 4710 | 24.1 | 15070_16186 | 1302 | 63.4 | 15137_15795 | 1626 | 43.0 |
| 14822_16188 | 1548 | 29.6 | 15095_16179 | 1284 | 18.4 | 12411_15101 | 5046 | 33.2 | 12826_15022 | 4710 | 23.1 | 1_15697 | 16860 | 12.2 | 15138_15794 | 1626 | 42.2 |
| 14820_16190 | 1548 | 29.3 | 15094_16180 | 1284 | 18.2 | 11174_16062 | 5322 | 5.2 | 9849_14705 | 8004 | 10.8 | 9596_15084 | 7878 | 11.0 | 11350_15130 | 6078 | 2.5 |
| 12858_16016 | 3684 | 5.4 | 13325_16155 | 3078 | 9.9 | 1_15133 | 17424 | 4.6 | 6119_15651 | 10788 | 8.9 | 9595_15085 | 7878 | 10.7 | 11351_15129 | 6078 | 2.5 |
| 15660_16130 | 768 | 2.1 | 15585_16181 | 792 | 8.7 | 11386_16138 | 5034 | 2.9 | 6118_15652 | 10788 | 8.8 |  |  |  | 11349_15131 | 6078 | 2.3 |
| 12238_14926 | 5394 | 1.0 | 15844_16138 | 576 | 4.9 | 12251_15531 | 4776 | 2.7 | 10536_14186 | 7836 | 2.3 |  |  |  |  |  |  |
|  |  |  | 15843_16193 | 522 | 4.3 | 11760_13130 | 7668 | 2.7 | 9417_11987 | 11154 | 2.2 |  |  |  |  |  |  |
|  |  |  | 13803_15515 | 3240 | 2.9 | 1_16099 | 16458 | 1.8 | 10537_14185 | 7836 | 2.1 |  |  |  |  |  |  |
|  |  |  | 13804_15514 | 3240 | 2.7 | 1_15847 | 16710 | 1.6 | 11987_14031 | 6540 | 1.5 |  |  |  |  |  |  |
|  |  |  | 13805_15513 | 3240 | 2.7 | 1_15745 | 16812 | 1.1 | 11986_14032 | 6540 | 1.5 |  |  |  |  |  |  |
|  |  |  | 14819_15633 | 2106 | 2.5 |  |  |  |  |  |  |  |  |  |  |  |  |
|  |  |  | 15696_16184 | 678 | 1.3 |  |  |  |  |  |  |  |  |  |  |  |  |
|  |  |  | 15695_16185 | 678 | 1.2 |  |  |  |  |  |  |  |  |  |  |  |  |

* Gray shading indicates cbVGs with adjacent break_rejoin positions that are likely to belong to the same species.

**Table S2:** **Variants detected in rS1, rS2, rS3 and rS4 stocks at Passage 1, 3 and 8.**

**A: rS1 stock.**

| Position | Ref | Alt | Alt_freq | | | Gene | REF_codon | REF_AA | ALT_codon | ALT_AA |
| --- | --- | --- | --- | --- | --- | --- | --- | --- | --- | --- |
|  |  |  | P1 | P3 | P8 |  |  |  |  |  |
| 20 | T | A |  |  | 0.93 |  |  | / |  | / |
| 24 | T | A |  |  | 0.19 |  |  | / |  | / |
| 24 | T | G |  |  | 0.09 |  |  | / |  | / |
| 58 | G | A |  |  | 0.44 |  |  | / |  | / |
| 274 | T | C |  |  | 0.08 | N | TTC | Phe | TCC | Ser |
| 2999 | A | G | 0.06 | 0.06 |  | P | AGT | Ser | GGT | Gly |
| 3684 | A | +G | 0.07 |  |  | P gene editing | | | | |
| 4444 | G | A | 0.81 | 0.82 | 0.63 | P | TAG | Stop | TAA | Stop |
| 6273 | G | A |  |  | 0.24 | F | GTG | Val | ATG | Met |
| 9996 | A | C | 0.82 | 0.83 | 0.63 | L | ATG | Met | CTG | Leu |
| 15250 | T | C |  | 0.30 | 0.21 | L | CTT | Leu | CCT | Pro |
| 15560 | T | C | 0.05 |  |  | L | CTT | Leu | CTC | Leu |

**B: rS2 stock.**

| Position | Ref | Alt | Alt_freq | | | Gene | REF_codon | REF_AA | ALT_codon | ALT_AA |
| --- | --- | --- | --- | --- | --- | --- | --- | --- | --- | --- |
|  |  |  | P1 | P3 | P8 |  |  |  |  |  |
| 20 | T | A |  |  | 0.92 |  |  | / |  | / |
| 24 | T | A |  |  | 0.82 |  |  | / |  | / |
| 58 | G | A |  |  | 0.07 |  |  | / |  | / |
| 3684 | A | +G | 0.08 |  |  | P gene editing |  |  |  |  |
| 7864 | A | G |  |  | 0.65 | HN | CAA | Gln | CGA | Arg |
| 8353 | A | G |  |  | 0.09 | HN | AAA | Lys | AGA | Arg |
| 9325 | C | A |  |  | 0.08 | Between HN/L | | | | |
| 15719 | A | T | 0.16 | 0.16 | 0.23 | L | ATA | Ile | ATT | Ile |

**C: rS4 stock.**

| Position | Ref | Alt | Alt_freq | | | Gene | REF_codon | REF_AA | ALT_codon | ALT_AA |
| --- | --- | --- | --- | --- | --- | --- | --- | --- | --- | --- |
|  |  |  | P1 | P3 | P8 |  |  |  |  |  |
| 20 | T | A |  | 0.19 | 0.72 |  |  | / |  | / |
| 24 | T | A |  | 0.09 | 0.75 |  |  | / |  | / |
| 58 | G | A |  | 0.05 |  |  |  | / |  | / |
| 2450 | A | C | 0.08 | 0.09 | 0.08 | eGFP | AGC | Ser | CGC | Arg |
| 2451 | G | A | 0.08 | 0.09 | 0.08 | eGFP | AGC | Ser | AAC | Asn |
| 3221 | C | T | 0.28 | 0.27 | 0.11 | P | CCT | Pro | TCT | Ser |
| 3684 | A | +G |  | 0.06 | 0.10 | P gene editing |  |  |  |  |
| 4094 | A | G |  |  | 0.07 | P | AAA | Lys | GAA | Glu |
| 6167 | C | T | 0.05 |  |  | F | ACC | Thr | ACT | Thr |
| 7372 | T | C | 0.06 | 0.06 | 0.15 | F | ATA | Ile | ACA | Thr |
| 7497 | A | T |  | 0.07 | 0.21 | Between F/HN |  | / |  | / |
| 7582 | A | C | 0.24 | 0.21 | 0.07 | Between F/HN |  | / |  | / |
| 8236 | C | T |  | 0.11 | 0.52 | HN | GCT | Ala | GTT | Val |
| 11128 | A | G |  |  | 0.07 | L | AAG | Lys | AGG | Arg |
| 12825 | A | G |  |  | 0.18 | L | ATC | Ile | GTC | Val |
| 13672 | A | G |  |  | 0.08 | L | GAT | Asp | GGT | Gly |

**D: rS6 stock.**

| Position | Ref | Alt | Alt_freq | | | Gene | REF_codon | REF_AA | ALT_codon | ALT_AA |
| --- | --- | --- | --- | --- | --- | --- | --- | --- | --- | --- |
|  |  |  | P1 | P3 | P8 |  |  |  |  |  |
| 20 | T | A |  | 0.19 | 0.46 |  |  | / |  | / |
| 24 | T | A |  | 0.08 | 0.49 |  |  | / |  | / |
| 449 | T | C |  |  | 0.07 | N | CCT | Pro | CCC | Pro |
| 2479 | C | G | 0.19 | 0.17 | 0.08 | eGFP | CCC | Pro | CCG | Pro |
| 3684 | A | +G |  | 0.06 |  | P gene editing |  |  |  |  |

* Gray shading indicates variants with a frequency higher than 0.5.

** DNA nucleotides (A, C, G, and T) are used instead of RNA nucleotides (A, C, G, and U) because next-generation sequencing outputs sequences in DNA notation.
